# Occurrence of larval fishes sampled by drifting light traps in the lower reaches of a South African estuary

**DOI:** 10.1093/plankt/fbad058

**Published:** 2024-02-02

**Authors:** Yanasivan Kisten, Michelle Kruger, Nadine A Strydom

**Affiliations:** Department of Zoology, Nelson Mandela University, South Campus, University Way, Summerstrand, P.O. Box 77000, Gqeberha 6031, South Africa; Department of Ichthyology and Fisheries Science, Rhodes University, Prince Alfred Street, Grahamstown, P.O. Box 94, Makhanda 6140, South Africa; Department of Zoology, Nelson Mandela University, South Campus, University Way, Summerstrand, P.O. Box 77000, Gqeberha 6031, South Africa

**Keywords:** gear selectivity, ichthyoplankton, light aggregation device, phototaxis

## Abstract

The tidal occurrence of larval fishes was investigated in the permanently open Kowie Estuary on the warm-temperate coast of South Africa. Larval fishes were sampled in the mouth region using two drifting light traps deployed on the ebb and flood tides every second night for two consecutive 14-day periods, coinciding with the dark moon phase. A total of 553 larval fishes were caught, representing nine families and 26 species, of which Blenniidae and Clupeidae dominated. The prevalence of different estuarine association fish guilds was also tide-specific. Marine and estuarine species, such as *Omobranchus woodi,* were more dominant during flood tides, while marine straggler species, such as *Sardinops sagax,* which are not dependent on estuaries, were dominant on the ebb tide. Marine estuarine-dependents were only present during flood tides, potentially indicating ingress and entrainment within the estuary. The results confirm that light trap catches yield a different composition of species compared to towed ichthyoplankton net studies. Additionally, drifting light traps allow for better targeting of species with a phototactic response and reduction of incidental catch. Consequently, a mixture of gear is encouraged for more comprehensive surveys of larval fish occurrence.

## INTRODUCTION

Estuaries are important nursery areas for young fishes worldwide (Potter *et al.*, 2015; Whitfield, 2019). Recruitment of marine larval fishes into temperate South African estuaries often occurs during the postflexion stage in larval development (Pattrick and Strydom, 2014; Whitfield, 2019). Postflexion larval fishes rely on both active swimming and passive tidal movements to aid their passage into nursery areas and maintain their position there (Whitfield, 1989; Aceves-Medina *et al.*, 2008; Pattrick and Strydom, 2014). The ebb and flood tides play an essential role in the exchange of larval fishes between the estuary and ocean, with significant energetic benefits occurring for larvae by moving with the flow (Trnski, 2001; Pattrick and Strydom, 2014).

International studies have mainly focused on the role of tidal stream transport on the recruitment of larval fishes into nursery areas and the influence of vertical and horizontal migrations on recruiting larvae in the estuary (Boehlert and Mundy, 1988; Trnski, 2001; Aceves-Medina *et al.*, 2008). South African research focusing on the use of the tidal cycle by larval fish in nursery areas is limited to a few studies on tidal exchange in specific local estuaries (Beckley, 1985; Whitfield, 1989; Strydom and Wooldridge, 2005; Pattrick and Strydom, 2014). The benefits of tidal transport are twofold, but it can also expose larvae to undesirable habitats (Boehlert and Mundy, 1988; Whitfield *et al.*, 2023). In order to prevent being swept out to sea on ebb tides and swept up into the estuary on flood tides, larvae need to move to the shallow marginal water of an estuary to avoid displacement and ensure entrainment within ideal areas of the estuary (Whitfield, 1989; Strydom and Wooldridge, 2005; Pattrick and Strydom, 2014). This larval fish recruitment or entrainment responses in estuaries can also be assisted by environmental cues such as temperature, salinity and turbidity (Boehlert and Mundy, 1988; Arevalo *et al.*, 2023; Whitfield *et al.*, 2023). Olfactory cues of chemicals from nursery areas and odors from further upstream are also integral for the recruitment and entrainment response in early-stage fishes in estuaries (James *et al.*, 2008; Whitfield *et al.*, 2023).

Standard methods used for assessing tidal exchange of small fishes include towed plankton nets (Strydom and Wooldridge, 2005), fine-meshed seine nets (Beckley, 1985) and stationary fyke nets (Pattrick and Strydom, 2014). It has been suggested that these methods are best used in combination to reduce individual gear-selective biases (Doherty, 1987; Choat *et al.*, 1993; Hickford and Schiel, 1999). The use of drifting light aggregation devices as an alternative or supplement to larval net studies has yet to be elucidated in estuaries worldwide.

Light traps function by exploiting the positive phototactic response displayed by most larval fishes (Doherty, 1987; Hickford and Schiel, 1999; Beckley and Naidoo, 2003). Sampling for larval fishes using light traps was pioneered in the 1980s to sample shallow weed-filled river waters where towing nets were not possible and has since been used to collect fish in various environments from shallow streams (Meekan *et al.*, 2000), estuaries (Strydom, 2003), to rocky reefs (Doherty, 1987; Choat *et al.*, 1993). However, light traps are still an under-utilized technique and may be especially useful for sampling shallow, slow-flowing waters and reducing damage to specimens (Doherty, 1987). This low utilization may be due to the major shortcomings of light traps compared to nets, which include inefficiency for accurate density measurements, low catch rates and the targeting of narrow larval sizes, depths and phototaxis sensitivity (Choat *et al.*, 1993; Hickford and Schiel, 1999; Meekan *et al.*, 2000).

In South Africa, the use of light aggregation devices to sample larval fishes is limited and was first conducted in the subtropical region by Beckley and Naidoo (2003), specifically in the Durban Harbor. The first study to use light traps as a sampling method in South African estuaries was conducted by Strydom (2003) and proved to be a successful technique in relatively clear shallow water and seagrass-dominated areas in an estuary. However, Thorrold (1992) found that static traps may exclude larvae that cannot enter the trap due to the velocity of the surrounding water. Consequently, the current study addresses this shortcoming by being the first to use drifting traps to assess the tidal exchange of larvae in an estuary worldwide.

The main aim of this study was to investigate the occurrence of larval fishes caught in drifting light traps at the mouth of the permanently open Kowie Estuary. It was hypothesized that the larval fish catch would vary between tides, and with associated environmental variability. This study also aimed to assess the suitability of a drifting light trap in tidal flows compared to other methods.

## METHODS

### Study area

**Fig. 1:**
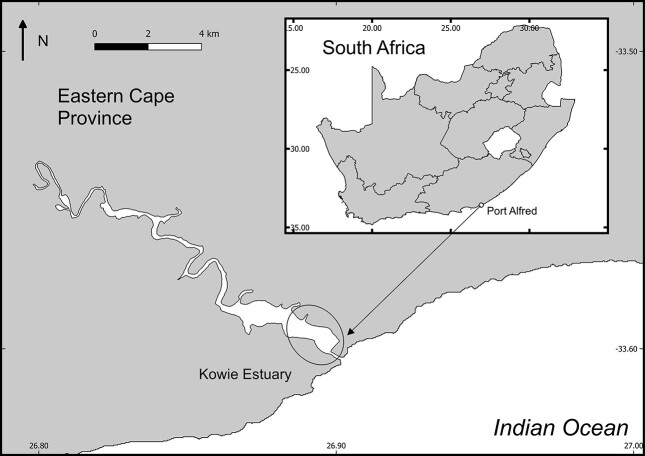
Geographical position of the Kowie Estuary in the Eastern Cape Province, South Africa. Light trap surveys were confined to the lower reaches and mouth of the estuary as indicated by the ellipse.

Larval fishes were collected from the mouth region of the permanently open Kowie Estuary (33°36′11” S; 26°54′10″ E) situated in Port Alfred on the southeast coast of South Africa (Fig. 1). The climate in this region is classified as warm temperate, with a bimodal rainfall pattern occurring in autumn and spring (Heydorn and Tinley, 1980). The lower reaches of the estuary are heavily impacted by anthropogenic activities, including the marina, which is characterized by artificial stone-packed walls. The river is ~70 km long, with an average depth of 2.75 m. The summer water temperatures range from 21 to 29°C, and winter temperatures range from 11 to 16°C (Hill and Allanson, 1971).

### Field sampling and larval identification

**Fig. 2:**
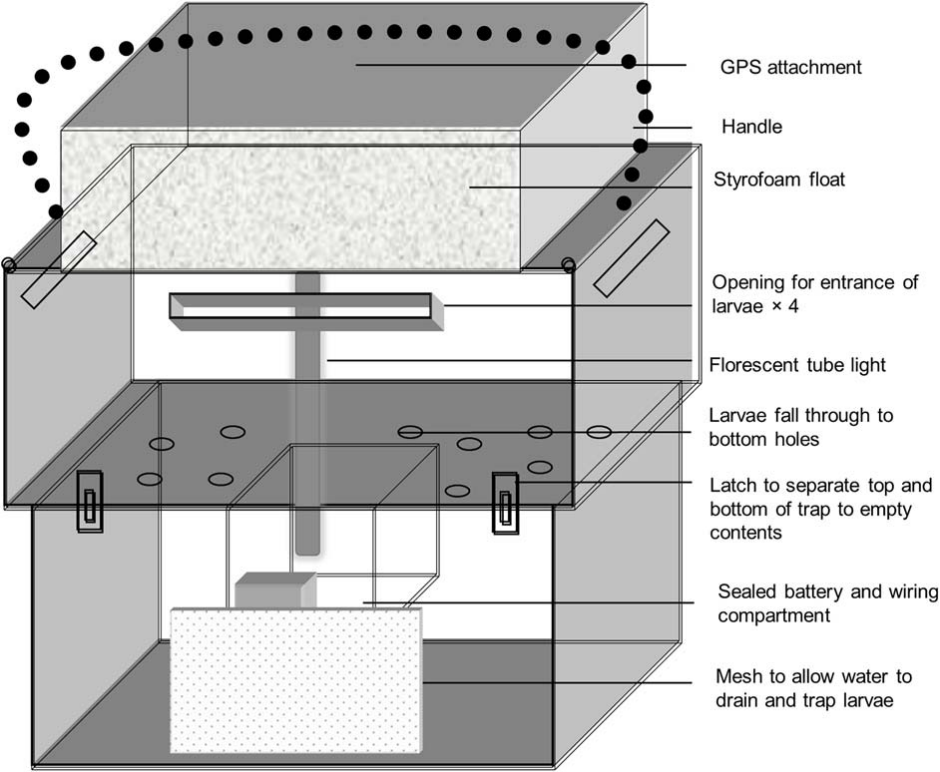
Diagrammatic representation of the drifting light trap used to catch larvae nocturnally in the Kowie Estuary in Eastern Cape, South Africa.

Sampling was conducted using two light traps designed for estuarine application in shallow water (Strydom, 2003). The Perspex trap housing comprised two main compartments (Fig. 2). The lower part of the trap contained the removable collection box where larvae were housed after capture. Each side of this removable box had smaller mesh windows to allow drainage when removed from the water. This section of the trap also contained another smaller inner box, which served as the waterproof area and housed a 12 V 12 amp.h^−1^ battery. The trap was switched on before deployment and then sealed (Strydom, 2003). The upper part of the trap contained the sealed 8-Watt fluorescent tube. Additionally, four sub-surface entrance slits were present on each side of the upper compartment. Larvae would enter through the slits in the upper compartment and be drained through numerous circular holes into the bottom compartment of the trap. The trap was floated by a large Styrofoam piece (Fig. 2).

Sampling commenced 2 hours after the turn of the ebb and flood tides and repeated four times per tidal state. Traps were deployed for 30 minutes at a time. All sampling occurred between sunset and sunrise and was conducted every second night over two weeks, coinciding with the dark moon period. Sampling was confined to the lower reaches and mouth region of the estuary. Two replicate sampling trips took place from 25 August to 6 September 2008 (survey one) and 23 September to 5 October 2008 (survey two). These dates were chosen to coincide with peak larval recruitment periods into estuaries in the region (Strydom, 2003).

A global positioning system was secured to the top of the trap to determine the distance traveled (m) as well as the moving average (km) and overall average (km) per 30-minute deployment. Additionally, at the start and end of each deployment, the physicochemical readings were recorded using a YSI 6600 Multiprobe to measure salinity, temperature (°C) and turbidity (NTU).

For deployment, the light trap was lowered off the edge of a boat in the middle of the channel and left to drift with the tide for 30 minutes at a time, ensuring minimal disturbance of the trap by the boat. The trap was then collected and emptied, with the contents preserved in a 10% buffered formalin solution after each 30-minute trapping interval.

In the laboratory, the larval fishes were separated from the remaining plankton and sorted, identified, counted, and measured. The larvae were identified to the lowest possible taxon, primarily following Smith and Heemstra (1995), Neira *et al.* (1998) and Leis and Carson-Ewart (2000). Larvae were measured to the nearest 0.01 mm using a dissecting microscope fitted with an eyepiece micrometer. For larger specimens, vernier calipers were used to measure body length (BL), defined as notochord length in preflexion and flexion larvae and standard length in postflexion and early juveniles (Strydom, 2003). Larval fish catches were expressed as catch per unit effort (CPUE), where unit effort was defined as the number of larvae trapped per 30-minute deployment. All positively identified larvae were then grouped into estuarine association fish guilds, according to Potter *et al.* (2015). Marine estuarine-dependents (MED) indicate marine species that rely on estuaries to complete their life cycles. Marine estuarine-opportunists (MEO) indicate marine species that use estuaries opportunistically but are not dependent on them. Marine stragglers (MS) indicate marine species that do not use estuaries in any significant way but can enter sporadically. Solely estuarine (SE) species occur only in estuaries, while estuarine and marine (E&M) species can occur in both E&M populations.

### Data analysis

All data were tested for homoscedasticity and normality for parametric test assumptions. Data that did not conform were analyzed using nonparametric tests. Environmental data, including salinity, temperature (°C) and turbidity, were tested for differences between ebb and flood tides and separated into surveys one and two. Biological data were also divided into surveys one and two and analyzed accordingly. Differences in species numbers and occurrence between the ebb and flood tide were investigated using one-way ANOVAs. Generalized Additive Models (GAMs) were used to test the effect of environmental variability on CPUE for the total catch and each of the five dominant species. A log-linked Poisson family distribution model was used due to the positively skewed and zero-inflated CPUE data. STATISTICA V10.0 was used for these univariate analyses. Unless otherwise stated, a significance level of *P* < 0.05 was used for all analyses.

## RESULTS

### Environmental variability

**Fig. 3:**
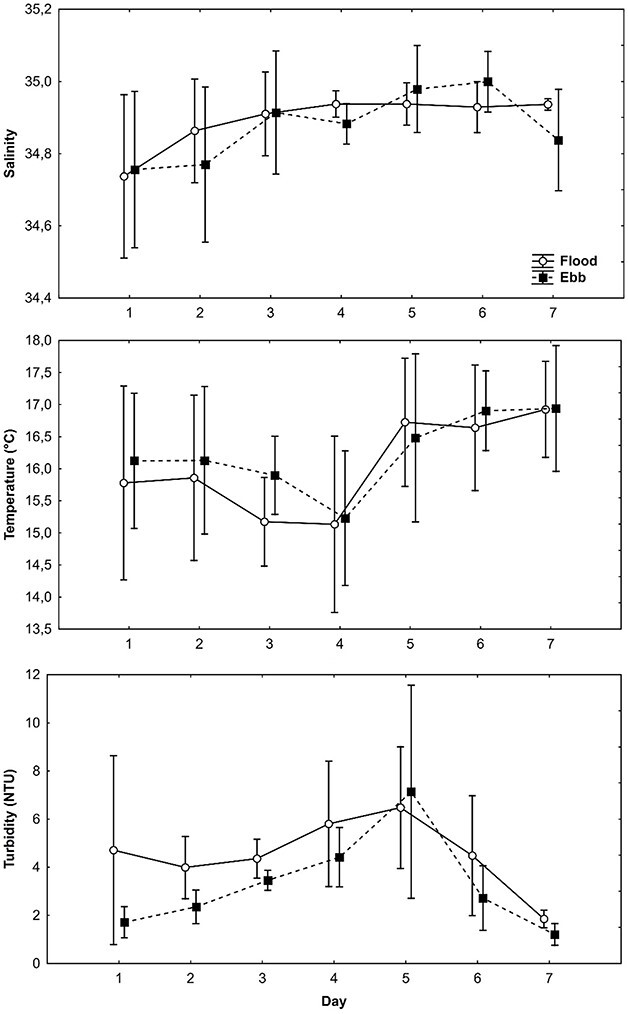
Mean environmental variability (salinity, temperature and turbidity ±95% confidence) in the lower reaches of the Kowie Estuary during two surveys in August and September 2008.

For survey one (August 2008), no significant difference between the ebb and flood tide occurred for salinity (F = 1.22, *P* = 0.27) and water temperature (H = 1.68, *P* = 0.20). However, turbidity was significantly higher during the flood than on the ebb tide (F = 5.08, *P* = 0.03). No significant differences were detected for environmental variables between the ebb and flood tides during survey two (salinity H = 2.85, *P* = 0.09, water temperature F = 0.36, *P* = 0.55 and turbidity F = 2.16, *P* = 0.15) (Fig. 3). For both survey one and two, the light trap seemed to cover more distance on the ebb tide, although not statistically significant (*P* > 0.05).

GAMs indicated that overall CPUE (all taxa) varied significantly with temperature, salinity and turbidity for many of the dominant species (Table I). Overall CPUE, *Omobranchus woodi*, *Sardinops sagax,* and *Clinus superciliosus* CPUE increased with decreasing salinity (Table I, *P* < 0.05, negative coefficient). Higher temperatures led to higher CPUE (Table I, *P* < 0.05, positive coefficient) except for *Pseudomyxus capensis,* which showed no effect (*P* > 0.05) and *Cirrhibarbis capensis,* which showed the opposite effect (Table I). Turbidity also significantly affected larval CPUE tested except for *C. supercilious* (Table I). Overall CPUE increased with turbidity, specifically for *C. capensis* and *P. capensis*, but CPUE decreased with turbidity for *O. woodi* and *S. sagax* (Table I).

**Fig. 4:**
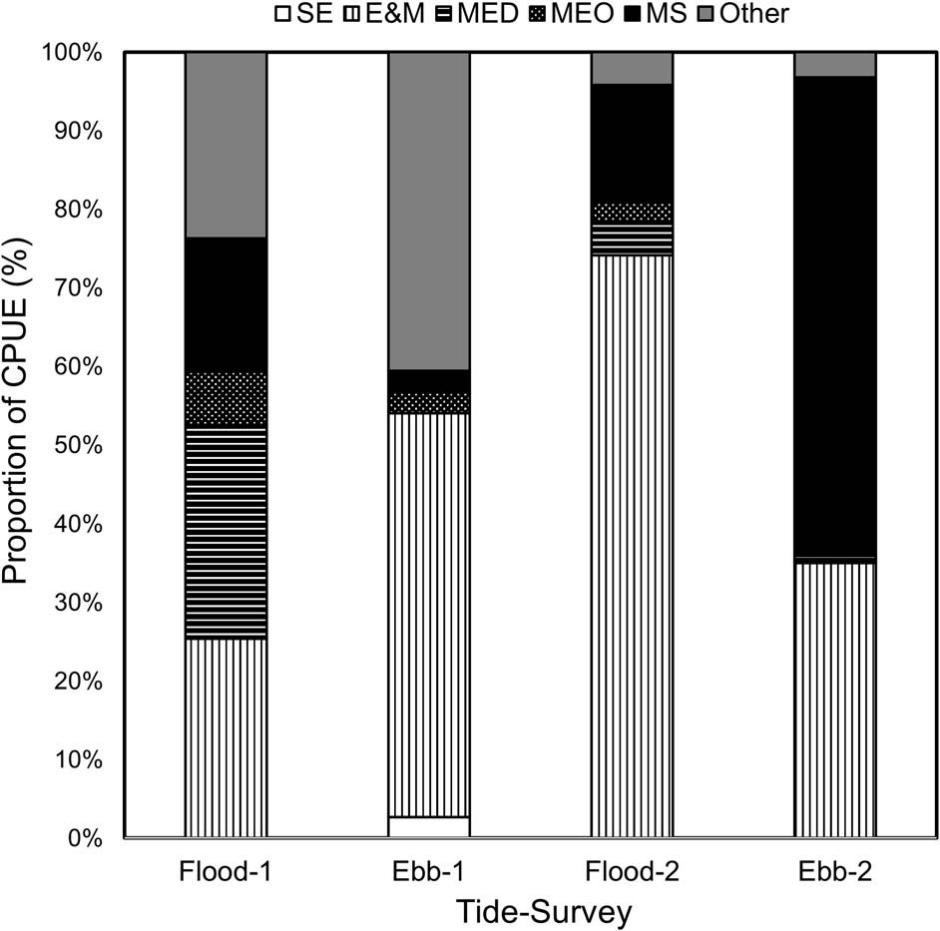
Percentage of larval fish guild representation (Potter *et al.*, 2015) at each nocturnal ebb and flood tide sampled during two light trap surveys (August–September 2008) in the mouth region of the Kowie Estuary. Guild abbreviations as per Table II.

**Table I:** Generalized Additive Model statistics (GAM, Log-linked Poisson) for larval fish CPUE versus environmental variables (temperature, salinity and turbidity) for all taxa and the dominant species in the Kowie Estuary lower reaches

CPUE	Model R^2^	Environmental Variable	Degrees of freedom	GAM coefficient	Standard error	*P*
Total	26.74	Temperature	4.13	0.32	0.06	< 0.005^*^
		Salinity	4.46	−1.26	0.34	< 0.005^*^
		Turbidity	3.90	0.01	0.02	< 0.005^*^
*Omobranchus*	52.88	Temperature	3.27	0.28	0.13	< 0.005^*^
*woodi*		Salinity	3.99	−2.37	0.46	< 0.005^*^
		Turbidity	3.43	−0.01	0.05	0.14
*Sardinops*	60.87	Temperature	3.67	0.10	0.20	< 0.005^*^
*sagax*		Salinity	3.92	−2.70	1.32	< 0.005^*^
		Turbidity	3.68	−0.14	0.06	< 0.005^*^
*Clinus*	39.53	Temperature	3.77	0.37	0.32	< 0.005^*^
*superciliosus*		Salinity	4.39	−0.58	1.65	0.01^*^
		Turbidity	2.65	−0.01	0.68	0.27
*Cirrhibarbis*	30.94	Temperature	3.94	−0.32	0.03	0.02^*^
*capensis*		Salinity	3.81	1.42	1.73	0.23
		Turbidity	3.55	0.07	0.11	0.02^*^
*Pseudomyxus*	57.31	Temperature	3.73	0.32	0.36	0.08
*capensis*		Salinity	3.46	1.15	2.50	0.09
		Turbidity	3.34	0.45	0.11	< 0.005^*^

Larvae were caught using nocturnally deployed drifting light traps. Asterisk (^*^) indicates a significant effect (*P* < 0.05).

**Table II:** Species composition, overall catch, catch per tide, mean CPUE, body length, developmental stage (dev. stage) and estuarine guild association for all fishes caught in the Kowie Estuary over the study period

Family	Species	Total	Ebb	Flood	Mean	Mean ebb	Mean flood	Body length (mm)		Dev.	Guild
		catch	catch	catch	CPUE	CPUE	CPUE	Mean	Range	stage	
Blenniidae	Blenny 1	1	1	0	0	0	0	10.2	10.2–10.2	Po	?
	Blenny 2	3	3	3	0	0	0	13.01	9.1–18.55	F, Po	?
	Blenny 3	8	2	6	0	0	0	9.1	8.5–9.1	F, Po	?
	*Omobranchus woodi*	231	65	166	8	2	6	16.8	10.5–27.8	Po	E&M
Clinidae	*Cirrhibarbis capensis*	23	10	13	0	0	0	26.9	21.1–31.9	Po	E&M
	*Clinus cottoides*	8	5	3	0	0	0	18.4	12.7–25.9	Po	E&M
	*Clinus superciliosus*	26	15	11	1	1	0	20	17.4–23.8	Po	E&M
	Clinid 1	1	0	1	0	0	0	15.16	15.16–15.16	Po	?
	Clinid 2	6	0	6	0	0	0	20	18.0–21.0	Po	?
	Clinid 3	2	0	2	0	0	0	17.6	17.0–18.2	Po	?
	Clinid 4	3	2	1	0	0	0	18.4	20.5–20. 5	Po	?
	Clinid 5	1	0	1	0	0	0	18.6	17.9–19.5	Po	?
	Clinid 6	2	1	1	0	0	0	16.5	16.5–16.5	Po	?
	Clinid 7	19	9	10	0	0	0	23.7	17.6–28.0	Po	?
Clupeidae	*Etrumeus whiteheadi*	3	1	2	0	0	0	14.2	5.1–23.6	F, Po	MS
	*Gilchristella aestuaria*	1	1	0	0	0	0	9.8	9.8–9.8	Po	SE
	*Sardinops sagax*	172	131	41	6	5	1	28.7	18.8–36.2	Po	MS
Gobiesocidae	*Eckloniaichthys scylliorhiniceps*	1	0	1	0	0	0	4.3	4.3–4.3	Pr	MS
Monodactylidae	*Monodactylus falciformis*	3	0	3	0	0	0	6.0	6.0–6.0	Po	MED
Mugilidae	*Mugil cephalus*	4	0	4	0	0	0	21.2	9.5–28.4	Po	MED
	*Pseudomyxus capensis*	17	0	17	1	0	1	11.7	5.2–13.7	Po	MED
Scorpididae	*Neoscorpis lithophilus*	3	1	2	0	0	0	12.2	11.7–12.6	Po	MS
Sillaginidae	*Sillago* sp. (likely *Sillago sihama*)	8	5	3	0	0	0	9.1	5.9–10.0	F, Po	MEO
Sparidae	*Diplodus capensis*	2	0	2	0	0	0	9.4	9.1–9.6	Po	MEO
	*Rhabdosargus holubi*	2	1	1	0	0	0	10.6	9.9–11.1	Po	MED
	*Sarpa salpa*	1	1	0	0	0	0	16.3	16.3–16.3	Po	MEO
	*Overall Catch*	553	254	299	20	9	11				

Pr = Pre flexion, F = flexion, Po = post flexion, SE = solely estuarine, E&M = estuarine and marine, MED = marine estuarine-dependents, MEO = marine estuarine opportunists, MS = marine stragglers

### Species composition

A total of 553 larval fishes were caught during the study period, representing nine families and 26 species. Blenniidae contributed 43% to the total larval fish catch, followed by Clupeidae at 32% and Clinidae at 17%. The dominant species were *O. woodi* (231 individuals; 42%) and *S. sagax* (172 individuals; 31%). Larvae of E&M fish species dominated the catches in the light traps deployed in the Kowie Estuary (52%). MS followed, contributing 32% to the total catch over the study period. Larval fishes that were MED, MEO, or SE contributed 6% toward the total catch. Regarding life stages, postflexion larvae dominated the catches, contributing 95.6% toward the overall catch, while preflexion and flexion larvae were recorded in insignificant numbers (3.9%).

### Larval tidal occurrence

Mean CPUE over the entire study period consisted of ~ 20 larval fish recorded per 30-minute deployment. A mean CPUE of 9 was recorded on the ebb tide, and a higher CPUE of 11 larvae was recorded on the flood tide. CPUE per 30-minute deployment was higher during survey two compared to survey one. However, no significant difference in CPUE was detected between the ebb and flood tide for both individual surveys one (H = 0.91, *P* = 0.34) and two (F = 0.03, *P* = 0.86). Survey one exhibited 21 species from 59 individuals on the flood tide and 13 species from 37 individuals on the ebb tide, while survey two exhibited 13 species from 240 individuals on the flood tide and nine species from 217 individuals on the ebb tide.

Larval occurrence during survey one did not exhibit clear trends in the difference of species belonging to estuarine dependence guild categories (Fig. 4). However, *P. capensis*, an estuarine-dependent catadromous mugilid, was predominantly present on flood tides (Table II). In September, the marine straggler *S. sagax* dominated the ebb tide, while the E&M *O. woodi* dominated the flood tide catches (Table II). Collectively (surveys one and two) on the flood tide, catches were dominated by category E&M, i.e. E&M larvae (64%). MS made up 52% of the catch on the ebb tide, followed by category E&M (38%) (Fig. 4).

Some species were tide-specific or present in different numbers on the ebb or flood tides (Table II). MED species, such as *Monodactylus falciformis* and *Rhabdosargus holubi,* and opportunists (MEO), such as *Diplodus capensis,* appeared predominantly on the flood tides during both surveys. However, MEO *Sarpa salpa* was recorded only on the ebb tide. This trend was also true for SE species for the ebb tide (Table II).

## DISCUSSION

This study aimed to examine the species dynamics of larval fish catches using drifting light traps to supplement towed-net assemblages and address the shortcomings of moored traps. It was found that the Family Blenniidae dominated the catches, followed by Clupeidae, Clinidae and Mugilidae. Regarding species dominance, the blenniid *O. woodi* contributed 42% toward the total catch, followed by the clupeid *S. sagax* (31%). A study using moored light traps by Strydom (2003) found a similar trend where the Blenniidae (45%) dominated the trap catches in the temperate, permanently open Swartkops Estuary. Other families recorded in the Swartkops light trap study included Atherinidae (22.4%), Mugilidae (22.4%), Gobiidae (9.8%) and Clupeidae (6%). Those species collected in the Swartkops light trap study were similar to the Kowie catches, with the same dominant species appearing in both estuaries using light traps (Strydom, 2003). Permanently open South African estuaries have higher species diversity than temporarily open/closed estuaries, and most often range from 21 to 37 of different species (Harrison and Whitfield, 2006a; Strydom, 2015). Therefore, both light trap studies reflect species and family numbers expected in a permanently open estuary.

*O. woodi* is an E&M species, with larvae present in both habitats (E&M), whereas *S. sagax* is a marine straggler species with no dependence on estuaries (MS) (Potter *et al.*, 2015; Whitfield, 2019). High catches of *S. sagax* on the ebb tide were unexpected; however, individuals likely entered the estuary on a diurnal flooding tide before sampling and were then flushed out. Beckley (1985) also found *S. sagax* on nocturnal ebb tides and suggested that these larvae may have been swept in on the previous flood tide and retained in the estuary until the following ebb state. Castro *et al.* (2005) indicated that high catches of engraulids and clupeids during the nocturnal ebb tide may be due to the avoidance of larger visual-based predators in Guanabara Bay. A previous tidal exchange towed-net study in Australia indicated no tidal direction preference for *S. sagax* larvae (Trnski, 2001). However, Trnski (2001) did find significant sensitivity of *S. sagax* to the lunar phase, which may indicate phototactic positivity.

*O. woodi* may have been returning to the estuary after being swept out as preflexion and was now at the stage ready to settle out of the plankton within the estuary (Whitfield, 2019). Beckley (1985) noted significant effluxes of *O. woodi* on the outgoing tide. Trnski (2001), amongst others, suggested that larvae originating from estuarine-spawned eggs, such as preflexion *O. woodi*, are generally more prevalent on the ebb tide, whereas marine-spawned larvae are typically more abundant on flood tides (Beckley, 1985; Whitfield, 1989; Strydom and Wooldridge, 2005). This appears logical for MED species such as *R. holubi* but does not take into account those larvae either passively trapped in tidal flux or those utilizing the water body for feeding purposes such as marine straggler *S. sagax*, as estuarine waters are rich in copepod prey, as was found in a concurrent study in the Kowie Estuary (Kruger and Strydom, 2011).

The dominant species recorded in the current study were compared to the top five species caught in the same estuary using towed nets in Table III. High catches of the blenniid *O. woodi* are often recorded in permanently open temperate estuaries in southern Africa (Strydom *et al.*, 2003; Strydom, 2015). However, other typical species expected in estuaries in this region (Table III), evident in previous towed net catches, such as the Gobiids, *Caffrogobius gilchristi* and *Psammogobius knysnaensis,* were not recorded in the Kowie Estuary light trap study and contributed 1% or less to the Swartkops light trap study (Strydom, 2003). Compared to the Kowie Estuary towed net study, which was run concurrently with this study (Kruger and Strydom, 2010), dominant families included gobiids, clupeids and blenniids. High gobiid larval fish catches are also prevalent in Australian net studies (Neira *et al.*, 1992; Neira and Potter, 1992). This is because gobiids and blenniids are typically benthic and only occur in the upper water column of estuaries at the preflexion larval phase and thus would not be reflected in light trap catches that require some active swimming into the trap, as light traps sample postflexion and presettlement larvae more effectively and towed nets sample preflexion larval fish more effectively (Doherty, 1987; Choat *et al.*, 1993). This also explains that despite the potentially large numbers of *O. woodi* and *C. gilchristi* preflexion larvae at the mouth (Kruger and Strydom, 2010), they were not present in the light traps in this study that require active movement. Additionally, some species of larval fishes may migrate vertically to the bottom during ebb tides to prevent advection out of the estuary and thus may be precluded from floating traps (Rowe and Epifanio, 1994; Churchill *et al.*, 1999). This would also be true for phototactic-negative species that may avoid light altogether.

**Table III:** Comparison of the percentage contribution of dominant species (top 5) toward the towed nets and light trap catches in the Kowie Estuary, as well as three other tidal mouth exchange studies in the warm-temperate region of South Africa

Family	Species	This study	Beckley (1985)	Whitfield (1989)	Strydom and Wooldridge (2005)	Kruger and Strydom (2011)
		Light traps	Towed net	Towed/push net	Towed net	Towed net
Atherinidae	*Atherina breviceps*	-	-	-	22	-
Blenniidae	*Omobranchus woodi*	42	-	8	-	-
	*Parablennius cornatus*	-	9	-	-	-
	Blenniid 1	-	-	-	-	14
Clinidae	*Clinus superciliosus*	5	-	-	-	-
Clupeidae	*Gilchristella aestuaria*	4	-	-	-	59
	*Sardinops sagax*	31	8	-	-	-
Gobiidae	*Caffrogobius gilchristi*	-	53	32	77	
	*Caffrogobius nudiceps*	-	-	-	-	9
	*Psammogobius knysnaensis*	-	-	27	44	8
Mugilidae	*Chelon richardsonii*	-	-	5	3	-
	*Pseudomyxus capensis*	3	-	-	-	-
Soleidae	*Heteromycteris capensis*	-	5	-	-	-
Sparidae	*Rhabdosargus holubi*		8	-	8	-
	*Spondyliosoma emarginatum*	-	-	16	-	-
Syngnathidae	*Syngnathus temminckii*	-	-	-	-	22
Other		15	17	12	6	8
	Total catch	553	6 623	-	1 529	1 497

Average salinity and temperature did not differ between the ebb and flood tide during the study period, potentially due to the lower reaches of the Kowie Estuary being marine-dominated and well-mixed. However, turbidity was found to be higher during the flood tides. Turbidity can be an important cue for recruiting larval fishes into estuaries (Harris and Cyrus, 2000; Kisten *et al.*, 2015; James *et al.*, 2022). However, the higher turbidity on the flood tide may have been linked to increased recruitment due to covariation with tidal-assisted ingress or olfactory attraction to cues from outgoing water on the previous ebb tide (James *et al.*, 2022). This was further evidenced by the environmental correlations in this study, with lower salinities and higher turbidities, typical of outgoing chemical cue-containing freshwater flow, resulting in higher CPUE (Table I).

Overall CPUE, and for many of the dominant species, generally increased with increasing turbidity, temperature and decreasing salinity. These are general trends for larval fish movement and occurrence documented in the literature (Harrison and Whitfield, 2006b; Kisten *et al.*, 2015; Strydom, 2015), but they can vary between species, as also seen in the current study. Higher flood tide catches of larval fishes in artificial channels in Australia were observed by Young and Potter (2003a, 2003b). With entrainment of the motile stages of MED within the estuary, lower light trap CPUE would coincide with the lower turbidity on the ebb tides, as shown in the current study.

The success of light traps as a sampling method depends on the ability of the larvae to observe the light, react to it, and then enter the trap (Doherty, 1987; Hickford and Schiel, 1999; Beckley and Naidoo, 2003). Therefore, those species that do not display a phototactic-positive response will be excluded from the catch. Also, the spectral range of the light presented must match the response of the targeted species (Gehrke, 1994). For example, species adapted to higher turbidities may exhibit increased responses to longer wavelengths of light (Gehrke, 1994), i.e. in the yellow–orange range. This evidence further supports the assumption that light traps sample specific species and sizes while excluding others.

Sampling selectivity, however, is not solely confined to light traps. Other sampling devices also show species, size and development stage selectively. A well-known weakness associated with towed-net sampling is the detection and avoidance of the net by larger, more agile larvae and hence the inefficient sampling thereof (Thorrold, 1992; Choat *et al.*, 1993; Trnski, 2001). Also, an added strength to using light traps is a reduction in structural damage of specimens compared to towed-net catches (Doherty, 1987); this may aid in identification or histological studies. However, it has repeatedly been established that light traps are more successful, yet biased, toward capturing and retaining larger-size larvae than plankton nets (Thorrold, 1992; Choat *et al.*, 1993; Hickford and Schiel, 1999).

It was also noted during sampling that larval fishes were seen actively swimming against currents in the mouth of the Kowie Estuary, possibly displaying rheotaxis to prevent being displaced by the flow of the ebbing tide. This phenomenon was later demonstrated by Pattrick and Strydom (2014) using alternate-facing fyke nets in the Swartkops and Sundays Estuaries. This would explain why some MED fishes in the current study would be absent in ebb tide catches as they move to the margins to stay entrained within the estuary (Pattrick and Strydom, 2014), rather than being attracted to light traps in the channel.

Postflexion stage larvae dominated the catches in the present study (95.6%); thus, most of the recorded larvae were potential settlers. This characteristic of light traps may be a partial reason for the atypical larval assemblage result compared to past tidal exchange investigations where MED and MEO species typically dominate the flood tide in estuary mouth regions, whilst SE species are typically more predominant on the ebb tide (Beckley, 1985; Strydom and Wooldridge, 2005; Pattrick and Strydom, 2014). Consequently, catch rates in light traps are much lower compared to towed nets, as seen in this (Table III) and other studies (Hickford and Schiel, 1999; Meekan *et al.*, 2000). This phenomenon is a major shortcoming of light traps as a sole method of determining larval fish assemblage structure as it targets certain larval stages and those species with phototactic responses, and predominantly samples ichthyoplankton in the upper water column. However, for the purpose of targeting phototaxis-positive postflexion larvae, the drifting light trap successfully excluded the non-motile stages that may enter moored traps due to incidental flow while allowing the time and space for the motile stages to enter (Thorrold, 1992; Meekan *et al.*, 2000). While the drifting light trap addresses the shortcomings of moored traps, concurrently measuring the adjacent flow may have further supported this conclusion. Increased repetition throughout the recruitment period may also be required as recruitment can be sporadic for some species. Nevertheless, this method does facilitate the sampling of the later larval stages of certain fish species that tend to show avoidance of conventional ichthyoplankton nets.

## CONCLUSIONS

In conclusion, this study demonstrated the occurrence of larval fishes caught using drifting light traps in the Kowie Estuary, a novel method that addresses the pitfalls of moored devices and supplements towed netting. Due to the tidal movement of water and larval fishes along with it, environmental variability also covaried strongly with CPUE. It was found that species composition differed compared to other studies with towed nets due to the targeted capture of phototaxis-positive larval fishes. The presence of MED on the flood tides may reflect a movement toward the margins at high tide to prevent advection back to the marine environment on the ebb tide. Thus, numerous studies, including the present one, have found that it is often best to combine various sampling methods to create a more accurate description of the larval assemblage in estuaries and thus minimize individual gear selectivity and, therefore, biased sampling (Doherty, 1987; Choat *et al.*, 1993; Hickford and Schiel, 1999). Drifting light traps, in particular, allow for a more targeted sampling of the late larval stages of phototactic fish species in estuaries.

## Data Availability

Data is available upon request.
